# Construct representation and definitions in psychopathology: the case of delusion

**DOI:** 10.1186/1747-5341-5-5

**Published:** 2010-02-25

**Authors:** Adriano CT Rodrigues, Claudio EM Banzato

**Affiliations:** 1 Health Sciences Centre, Federal University of Piauí - UFPI, Teresina - Piauí, Brazil; 2 Department of Psychiatry, Faculty of Medical Sciences, University of Campinas - UNICAMP, Campinas - São Paulo, Brazil

## Abstract

**Background:**

Delusion is one of the most intriguing psychopathological phenomena and its conceptualization remains the subject of genuine debate. Claims that it is ill-defined, however, are typically grounded on essentialist expectations that a given definition should capture the core of every instance acknowledged as delusion in the clinical setting.

**Objective:**

In this paper, we attempt to show the major limitations of the definition of delusion from a non-essentialist point of view.

**Method:**

The problem is analyzed within the framework of constructs and their translation into definitions. Different linguistic and epistemological perspectives that do concur when one deals with psychopathological phenomena are also considered.

**Results:**

The 'construct of delusion', rather than its clinical instances, is the reference in which its definition appears inept. Here we claim that the broad contextual and pragmatic bases that underpin the construct of delusion tend to be either overlooked or downplayed in the quest for a satisfactory definition of this phenomenon.

## Research

 

*"Whatever is made explicit, something is always left implicit." Tim Thornton *[1]

 

## Introduction

Since the 16^th ^century, delusions have been conceptualized as judgments, beliefs, or ideas that are pathologically false, erroneous, or impossible [2,3]. Currently, the most widespread definitions of delusion still rest upon this basis, with the addition of the external features put forward by Karl Jaspers in the early 20^th ^century, which would supposedly help us to recognize such phenomenon: its incorrigibility and the certainty with which it is held. Along these lines, in the DSM-IV [4], and very similarly in the ICD-10 [5], delusion is defined as:

"...a false personal belief *[falsity] *based on incorrect inference about external reality and firmly sustained *[conviction] *despite of what almost everyone else believes and despite of what constitutes incontrovertible and obvious proof or evidence to the contrary *[incorrigibility]*. The belief is not one ordinarily accepted by other members of the person's culture or sub-culture". *Authors' brackets*.

However, each of these features could be seen as a defeasible element in the definition of delusion. Accordingly, some authors emphasize that delusional statements are occasionally found to be true [6-8]. For example, someone who firmly believes they are secretly watched by authorities, and who finds evidence of it in every trivial fact of daily life, may be coincidently found to be under actual investigation. Given the coincidental character of circumstance, this belief matches empirical truth, yet its delusional nature is still obvious. Similarly, the literature provides ample evidence that delusions are held with various degrees of conviction and incorrigibility [9-19]. For example, we could present a patient that believed to have a key role in an ongoing galactic war, but never held this belief with absolute conviction. The same patient believed he would live for more than a thousand years unless he committed suicide, but was equally unsure about it. Another patient, after two years with a persecutory delusion refractory to antipsychotics, and in response to her family's eager efforts to prove it false, finally yielded: "Well, I still feel as if someone was watching me and video recording everything I do, but I must agree that this is not possible...this is not actually going on". So, while the features of falsity, conviction and incorrigibility are not singularly necessary to make a delusion, it is also recognized that they are not sufficient for such a diagnosis [6,9,20-22]. In this way, we note that mystical and ideologically grounded ideas are often false, held with conviction and incorrigibility, but at the same time clinically viewed as non-delusional.

Indeed, although there are other refutations of the standard definition of delusion in the literature, most are prompted by the fact that *'falsity'*, *'conviction' *and *'incorrigibility' *are neither restricted to delusional propositions nor always present in them. However, a closer look at how these criticisms are typically formulated suggests that they fail to recognize the different conceptual issues that are involved. Furthermore, they are seemingly embedded in an anachronistically essentialist view of psychopathology [23]. We argue that the vagary of the definition of delusion needs to be considered both in a practical, as well as more strictly conceptual level, so as to enable a non-essentialist (or at least non-anachronistically essentialist) perspective to be considered. We believe that unless these particulars are adequately recognized and examined, it will not be possible to develop and hold a proper conceptualization of delusions that is relevant to cross-cultural clinical practice.

## The perils of ambiguous criticisms

By and large, scientific definitions aim to provide the best possible depiction of the set of objects to which a term refers, and in this way establish a standard for further identification of similar objects. An adequate approach to the flaws ascribed to the definition of delusion requires that both of these roles are recognized, and also necessitates acknowledgement that these functions are relatively independent from one another.

A definition may be a reliable and accurate means to identify things that belong to a certain class of objects and, still, may be a poor portrayal of the class to which that object belongs. Operational definitions employed in scientific research (and other practical purposes) often exhibit this limitation. So, for example, an operational definition of diabetes based upon blood glucose levels may accurately identify individuals to treat, or to allocate in a research group. On the other hand, it neglects information basic to the understanding of diabetes such as its pathology and clinical implications. The same is true when depression is defined according to a score above a certain threshold on the Hamilton Rating Scale for Depression. Arguably, the opposite may occur as well, and a depiction of the meaning of a certain term often fails to provide accurate identification of how it should be applied and/or used. Thus, while Bleuler's characterization of schizophrenia could be considered compelling, it would conceivably offer poor guidance to decision-making in some concrete clinical situations [24].

This dissociation basically reflects the different requirements involved in the formulation of a given definition. In order to be informative, a scientific definition must offer a good description of the circumstances in which a certain term is used (as narrow, wide, clear-cut or loose as they may be). On the other hand, its utility in identifying cases that fit into this set of objects requires that the definition be based on features that, besides being conspicuous and objective, are also as exclusive and as ubiquitous as possible among the members of that class of objects. In other words, as a tool to identify cases that pertain to a certain class, a definition should emphasize features that establish reliable and precise limits for that class.

Admittedly, certain classes of objects can be properly described by means of features of this latter type, and so both aspects of a definition can be assumed in such situations. Nevertheless, when focusing upon a non-discrete class of objects, or when a definition has eminently evaluative underpinnings or effect(s), an indisputable and straightforward identification of its instances is necessary, yet often remains artifactual. In such situations, no matter how descriptively sophisticated the definitions of these classes, no single criterion can legitimately provide a sharp distinction between cases and non-cases. Indeed, definitions would at best be able to reproduce the primarily fuzzy character of those classes, and the categorization of these phenomena would fit better within a prototypical rather than a traditional model [25]. Psychiatric disorders seemingly belong to this sort of phenomena, as no criteria (including criteria external and independent to their current definitions) have yet been shown to draw zones of rarity between one another or between them and normality [26]. Although the work of Patil and Giordano is attempting to substantiate valid, eidetic definitions [23], in these situations, definitions typically describe clusters of features that, because of their relevance, are granted a special place in the conceptual schema. Accordingly, such definitions often describe the prototypical case (i.e.- the ideal case) of each class, and decisions regarding other particular cases reflect their placement along a graded continuum of similarity to these prototypes.

Since the roles of definitions depend on distinct and often independent attributes, we posit that any criticism that could be made to either of these roles should require equal specificity. As previously mentioned, criticism of the definition of delusion commonly identifies features that are neither necessary nor sufficient to identify a case of this phenomenon [7-9]. Although this disapproval highlights the *practical definition *of delusion, a careful inspection may suggest that this is not solely the concern. The accuracy of that definition to distinguish what is delusional from non-delusional is rarely (if ever) referred to as a matter of degree - as might be expected according to a strictly practical perspective. Furthermore, there is little mention of evidence (or even prediction) that the strict implementation of the definition would lead to a significant number of errors or dubious clinical diagnoses. While the mis-match between the definition and certain clinical instances of delusion has been convincingly demonstrated on empirical grounds, these data are typically omitted in any such criticism of the definition. The way that these asymmetries are usually treated seems to reflect a deeper concern about the definition of delusion.

A more basic conceptual issue is seemingly inferred by critics. Mainly, they appear to believe that if the identification of delusion is sometimes hampered by its definition, this is because the definition poorly depicts how this phenomenon is actually presented and experienced. In other words, it is supposed that an intuitive, but more appealing notion of delusion rivals the standard definition, and this might be of greater psychiatric utility in identifying delusional cases. As a consequence, these critics nourish the expectation that unveiling such an underlying notion of delusion would then enable us to re-shape the definition to more accurately describe the phenomenon and clinical event.

At this point the problems of ambiguous criticism of the definition of delusion become apparent. As we have seen, reliable and accurate identification of the instances of a certain class of phenomena requires a definition to emphasize conspicuous and objective features. These features should allow us to draw precise limits between one phenomenon (e.g.- delusion) and other classes of phenomena. Thus, the accuracy of any tentative new definition of delusion may not be guaranteed even by the most painstaking reappraisal of our underlying view of this phenomenon. It would also depend on whether or not such view is committed to ideals of objectivity and discreteness. This cannot be taken for granted. It is conceivable that delusions are identified on a case-to-case basis and that the overall notion of delusion merely reflects the 'tendency' of various criteria to group together. Thus, perhaps a prototypical approach is spontaneously and implicitly at play when we deal with delusion, even if we make some essentialist assumptions regarding its definition [23].

Unfortunately, this tends to be overlooked in most of the approaches to the subject. Indeed, Gipps and Fulford (2004) provide exception in recognizing that perhaps an essential definition of delusion remains intangible - at least according to prior conceptualizations of what an 'essence' of psychiatric disorder might be [9].

Of course, since the discreteness of this phenomenon is not wholly rejected, Gipps and Fulford (2004) are probably right when they suggest that the pursuit of the 'actual' meaning of the term delusion should not be prematurely abandoned. However, we choose to consider what sort of legitimate criticism to the definition could be made (as a non-discrete class of phenomena) and we will try to show how a non-essentialist definition of delusions could be advanced.

## The complex construct of delusion

If no clear-cut limits seem to exist between delusions and other phenomena, then requiring a given definition to provide clinically relevant differentiation with precision is not reasonable. Even within a non-essentialist framework, concerns about whether the definition of delusion adequately depicts this class of phenomena remain genuine. If delusions are not assumed to have an essence, our concerns here are whether a definition then neglects important yet tacit aspects of this phenomenon. Whereas an essentialist perspective provides a view of delusion that is grounded to discrete features, a non-essentialist orientation may require a more complex construal of the meaning of delusion(s) as phenomenon, experience, and clinical event. We argue that a pragmatic and contextually embedded hypothesis of construct formation may provide a valuable framework to understand the complex meaning of the term 'delusion'.

Accordingly, whether or not the definition of a given term is well established, our views upon the construct to which it refers are affected by a variety of explicit and implicit information. Therefore, in addition to those features already addressed as regards the definition of delusion, other elements could be incorporated into an understanding of this phenomenon. These could include theoretical formulations, findings of empirical studies, as well as significant aspects learned from clinical practice [27]. Indeed, in some situations, the relevance of these factors could equal and/or perhaps supercede less socio-culturally embedded features. Accordingly, both in public and scientific language, the meaning of the term 'delusion' - that is, what we think delusions *are *- can be seen as a changing notion. In this way, elements could affect our views of delusion in an almost completely implicit way. Wittgenstein's ideas about the meaning and the uses given to terms of language may provide us a useful step to understand how implicit information could be integrated to broad concepts about delusion.

To Wittgenstein, the asymmetry between the meaning and definition of a term was clear and fundamental; the meaning of a term is not given by those features included in its definition, but by each of the situations in which it is employed. In addition, the decision as to whether the same label should be applied to other cases would depend on their degree of resemblance to the previously known uses of that term, possibly engendering new references to its meaning [28]. Learning the situations in which a term is used is a continuous process that recognizes both theoretical and practical influences. But in this case, meaning is embedded in a much more deeply contextual frame of reference. Each of the complex circumstances in which a term is employed becomes one of its particular meanings, as well as a distinctive *'unit' *of reference to the identification of similar cases.

Accordingly, the overall presentation of those situations (i.e.- the cases as wholes) could be as important as any specific, defining features. But although each of the cases in which a term is used may have constituent elements, those cases could not be described as a simple sum of their parts. Within these wholes, relevant aspects of our way of dealing with terms are kept implicit. This view is in agreement with the fact that the identification of instances of a phenomenon may take place without the examiner's awareness of using explicit criteria. It may also explain why individuals are often unable to define a phenomenon that they have correctly identified [7].

In this light, it is worthwhile to illustrate the sort of contextual influences that might often be neglected as regards the characterization and definition of delusions. Since a phenomenological approach to psychopathology gives special attention to contextuality, it offers an excellent epistemological framework for the conception of language, as described above. According to this perspective, delusions could neither be conceived apart from other signs and symptoms, nor from the whole clinical scenario in which they occur. Indeed, there are aspects inherent to the situations in which delusions are (claimed to be) present, but that are not properties of delusion itself, and these may possibly play a role. The attunement and overall interaction between the patient and the observer (or the community), for instance, could provide a context for both identifying delusions, and affording meaning to this term. Taking delusion to be the detachment of an individual from the system of relations that regulates meanings and the use of concepts [29], a quasi-solipsism [30,31] and/or a failure to share common sense [32], all may suggest central elements in the identification and values important to this term (and concept). Following these authors, we argue that a radical rupture between an individual and the socio-linguistic community in which they are nested (i.e.- an interface phenomenon), is a core aspect of delusion.

Yet, while an overall impression of disconnection between 'the deluded' and other individuals may be a valuable (albeit hypothetical) construal, it does not mean that we should disregard the role of those features currently included in its clinical definition [33,34]. To the contrary, departing from the standard definition of delusion would be to err in the opposite direction. As noted by Thornton (2006): *"... that tacit knowledge is a necessary element of scientific judgment does not undermine a practical use for some codifications. But it does suggests a principled limit to the ambition to codify all that is involved in having good judgment" *[1]. Falsity, incorrigibility, and conviction also are important to many aspects of the term delusion; thus, they must be allowed some equal relevance to the meaning of the term. These features, and a detachment from common sense may occur simultaneously, and each may exert an independent role in certain cases and, in this way, determine distinct subsets of delusion.

It is not expected that the linguistic and epistemological models suggested here that relate to the interpersonal facets of delusion should be considered to truly 'unmask' the phenomenon. Rather, they should be best understood as a common-sense and spontaneous way to deal with delusions, if not psychiatric phenomena in general, that is complementary to the explicit and systematized way these events are approached within naturalistic scientific models. There is a dialectical relation between these perspectives, and so we pose that the initial tension between their supposedly competing views can be articulated in a more sophisticated and possibly more fruitful definition of delusion.

Although 'incomprehensibility' is currently excluded from the standard definition of delusion, the ability of this construct to discriminate 'delusion-like ideas' and 'true delusions' [6] makes it relevant. It must be noted, however, that the idea of a 'detachment from common-sense' departs from the notion of incomprehensibility, both in its Jaspersian sense, and in the non-referential view proposed by Heinimaa [35]. The lack of attunement between deluded individuals and others, as previously discussed, is more broadly woven in the lived situation of the person/patient, such that physiognomy, speech peculiarities, ideo-affective coherence, overall behavior, thought style, reactions to the observer, and so on, all can - and likely are - critically involved. For example, the emotional response of a patient to an odd belief she holds may have a fundamental influence over our clinical intuition regarding her 'lack of attunement' to the socio-linguistic community. But at the same time as this patient's emotional response could be a recognizable feature of detachment from common-sense (as perceived by others), our impressions of the latter could not necessarily be reduced to, or explained by the former.

## Conclusion

We have argued that if delusions are supposed to constitute a non-discrete class of phenomena, then it is not reasonable to expect that any current definitions will allow an accurate distinction between its instances and those of other phenomena. On the other hand, there is legitimate concern as to whether its definition provides adequate description and meaning of the term delusion, even if non-essentialist assumptions prevail. In addition, we opine that our views on delusion are amenable to continuous change based upon new practical and theoretical data. Thus, a certain gap could exist between an established definition of a term, and its ever-evolving meaning. The current, most widespread definition of delusion - grounded on explicit and supposedly more objective features - possibly neglects critical aspects of this class of phenomena.

So while the contemporary scientific approach to psychopathological phenomena adopts an objective and descriptivist attitude, part of our view on delusion is embedded in a more linguistic and therefore different epistemological framework. In this way, the term delusion finds meaning in the situations in which it is used; it exposes the relevance of non-analytical processes in identification of cases by not focusing solely on the phenomenon itself, but by also attending to context, as a whole. We believe that the schism between the deluded individual and her socio-linguistic community - widely reported within phenomenological psychopathology - is an implicit, but relevant element of those clinical scenarios in which we can identify delusions.

Finally, the expected consequences of this discussion should by no means be taken as a prompt to urgently change the definition of delusion, as a balance between the benefits and limitations of any proposed definition must be considered at all times, bearing in mind its intended purposes and goals. Yet, we maintain that it is important to acknowledge the complex influences over our views of psychopathological phenomena, recognizing how these may affect - and be affected by - clinical expertise, and therefore should all be taken into account so as to open rich paths for future research.

## Competing interests

The authors declare that they have no competing interests.

## Authors' contributions

AR prepared the first draft of the manuscript. CB reviewed the first draft of the manuscript and made significant amends in regard of its content and structure. They thoroughly discussed the ideas within the paper and agreed in the final version of the manuscript.

## Authors' information

AR has completed his psychiatric training and got his Master's in Science from the University of Campinas - UNICAMP (Brazil), where he currently carries on his PhD research. He is an Assistant Professor of Psychiatry at the Federal University of Piaui - UFPI (Brazil).

CB is a psychiatrist and doctor of philosophy. He is currently Associate Professor of Psychiatry at the Faculty of Medical Sciences of the University of Campinas - UNICAMP (Brazil).
